# Robust, Scalable, and Triboelectric‐Responsive Superhydrophobic Coating for Versatile Smart City Applications

**DOI:** 10.1002/smsc.202500387

**Published:** 2025-09-13

**Authors:** Mingrui Wang, Ziyi Dai, Lining Zhang, Tian Tang, Kai Qian, Lihua Tang, Kean C. Aw, Zhiyi Wu

**Affiliations:** ^1^ Department of Mechanical and Mechatronics Engineering The University of Auckland Auckland 1010 New Zealand; ^2^ Beijing Institute of Nanoenergy and Nanosystems Chinese Academy of Sciences Beijing 100083 China; ^3^ School of Integrated Circuits Shandong University Jinan 250100 China

**Keywords:** large‐scale, self‐cleaning, smart city, superhydrophobic coating, triboelectric nanogenerators

## Abstract

Self‐powered sensing networks are essential for smart city infrastructure, with triboelectric nanogenerators (TENGs) emerging as a promising technology for distributed sensing and energy harvesting. However, widespread TENG implementation is hindered by moisture‐induced charge dissipation in urban environments. While superhydrophobic surfaces can mitigate this issue, existing coatings lack sufficient triboelectric properties for effective charge generation, while suffering from mechanical fragility that limits practical deployment. Herein, a triboelectric‐responsive superhydrophobic coating (TRSC) is reported that achieves thorough drying within 90 s at room temperature with remarkable cost‐effectiveness (<US$1 m^−2^). The coating exhibits consistent superhydrophobicity (contact angle 157°) and stable electrical output after 500 cycles of mechanical abrasion, tape‐peeling, and compression tests. When deployed as smart city sensors, TRSC enables solid–solid contact sensing for traffic monitoring, solid–liquid interfacial energy harvesting from raindrops, and noncontact sensing for human activity detection. The coating maintains performance under 99% relative humidity and shows excellent adhesion on various substrates regardless of surface roughness, microstructure, and geometric complexity. Compatible with automatic spraying systems and conventional equipment, this coating strategy enables large‐scale manufacturing to transform existing urban infrastructure into smart sensing networks, marking a significant step toward practical smart city implementation.

## Graphical abstract

A triboelectric‐responsive superhydrophobic coating is presented to overcome moisture‐related performance decline in nanogenerators. Cost‐effective, self‐cleaning, and mechanically robust, this coating maintains stable operation in high humidity. By allowing for mass production of self‐powered sensors, it provides a practical solution for smart city applications such as traffic monitoring and human activity tracking.

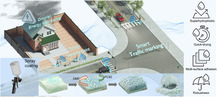

## 1 Introduction

Smart cities rely heavily on massive data collection and analysis to achieve efficient resource allocation and intelligent management.^[^ ^1^ ^]^ This digital foundation demands extensive deployment of distributed sensing networks, which serve as the fundamental infrastructure for real‐time monitoring and data acquisition across various urban sectors, including transportation, security, and environmental protection.^[^ ^2^, ^3^, ^4^ ^]^ However, the conventional approach of integrating sensing functionalities into urban infrastructure not only requires complex wiring systems and substantial modifications to existing structures, but also raises esthetic concerns with visible sensing components disrupting urban landscapes.^[^ ^5^ ^]^ This dual challenge has sparked interest in additive manufacturing for sensing technologies that can transform existing nonintelligent infrastructure into smart components through unobtrusive surface modifications. Among various strategies, self‐powered sensing systems have emerged as a promising solution, eliminating the need for external power sources and complex integration processes while maintaining architectural esthetics.^[^ ^6^ ^]^ Triboelectric nanogenerators (TENGs), operating through solid–solid contact, solid–liquid interfaces, and noncontact mode, can simultaneously harvest mechanical energy and generate sensing signals while being seamlessly integrated into urban surfaces.^[^ ^7^ ^]^ These diverse working modes facilitate energy harvesting and signal generation in transportation monitoring, security surveillance, and environmental sensing, enabling coating‐based TENGs for constructing esthetically integrated digital infrastructure in future cities.^[^ ^8^ ^]^

TENGs operate based on the coupling effect of contact electrification and electrostatic induction between two materials with different electron affinities.^[^ ^9^ ^]^ During operation, periodic mechanical movements induce charge transfer at the interface of contact materials, followed by the redistribution of electrostatic charges, generating alternating current output.^[^ ^10^ ^]^ In a typical configuration, dielectric materials such as nylon or polytetrafluoroethylene (PTFE) serve as triboelectric layers paired with metal electrodes, generating electrical output through periodic contact‐separation or sliding motions.^[^ ^11^ ^]^ The versatility of this mechanism enables TENGs to operate in multiple modes, including vertical contact‐separation, lateral sliding, single‐electrode, and freestanding modes, each suited for different application scenarios. Recent advances have demonstrated the potential of TENGs in smart city applications through various device configurations.^[^ ^12^, ^13^ ^]^ For instance, Deng et al. demonstrated intelligent tiles by integrating electrodes with ceramics through temperature gradient sintering, enabling distinct signal recognition for different motion patterns.^[^ ^14^ ^]^ Li et al. developed wind‐driven TENGs for agricultural applications, powering night‐time plant illumination and soil temperature monitoring.^[^ ^15^ ^]^ Yang et al. reported self‐powered traffic sensors fabricated from electrospun composite nanofibers for intelligent transportation monitoring.^[^ ^16^ ^]^ The expanding scope of TENGs into diverse applications, including acoustic energy conversion, smart security systems, and sustainable energy solutions from waste materials^[^ ^17^, ^18^, ^19^, ^20^ ^]^ underscores the urgent need to bridge the gap between laboratory demonstrations and real‐world deployment. As research moves toward practical implementation, stability emerges as a critical challenge for TENGs, particularly in complex urban environments.^[^ ^21^ ^]^ Especially, the presence of moisture significantly impacts TENG performance, as water molecules can cause swelling of the sensing substrate, leading to response lag, impedance attenuation, and electrostatic shielding.^[^ ^22^, ^23^ ^]^ Moreover, humidity‐induced charge dissipation severely compromises the surface charge density, which is crucial for TENG operation. Traditional encapsulation strategies often result in bulky devices requiring complex mounting procedures and structural modifications, limiting practical deployment.^[^ ^24^ ^]^

To address these environmental challenges, particularly moisture interference and dust accumulation, superhydrophobic surface modification has emerged as a promising strategy for TENG protection and performance enhancement.^[^ ^25^ ^]^ Superhydrophobic surfaces, characterized by water contact angles exceeding 150°, not only effectively repel water droplets but also demonstrate self‐cleaning capability against solid contaminants.^[^ ^26^ ^]^ The working mechanism of superhydrophobic TENGs relies on two key features: the reduced surface energy minimizes charge neutralization by environmental moisture, while the hierarchical micro/nanostructures increase the effective contact area and surface charge density, collectively enhancing charge transfer efficiency.^[^ ^27^, ^28^, ^29^ ^]^ Early explorations primarily focused on adding supplementary superhydrophobic layers onto existing triboelectric materials through simple spray‐coating methods. While these additional layers achieved water contact angles above 160° and demonstrated improved TENG electrical output, the interface between the superhydrophobic and triboelectric layers could potentially compromise long‐term durability.^[^ ^30^ ^]^ In contrast, the direct integration of superhydrophobic properties into the TENG material itself enables stable operation in complex environments through enhanced charge retention and environmental tolerance without interface concerns. Various fabrication strategies have been developed to create superhydrophobic protective layers, including bottom‐up approaches such as electrochemical deposition that allow precise control of surface structures^[^ ^31^ ^]^ and top‐down methods like plasma etching that offer rapid surface modification.^[^ ^32^, ^33^ ^]^ These modified TENGs exhibit excellent resistance to various environmental factors, including moisture, dust, and biological contamination, broadening their application scenarios.^[^ ^34^ ^]^ However, the implementation of superhydrophobic TENGs faces several critical limitations: 1) the hierarchical micro/nanostructures essential for superhydrophobicity are inherently fragile, making them susceptible to mechanical damage during practical use; 2) the traditional fabrication methods for superhydrophobic surfaces often involve complex processes or expensive materials, limiting their scalability; and 3) many existing approaches are substrate‐specific, restricting their application across diverse urban surfaces with varying roughness and geometries. These limitations highlight the need for a more robust and versatile approach that integrates triboelectric and superhydrophobic functionalities within a single material system while enabling practical large‐scale deployment.

Herein, we report a triboelectric‐responsive superhydrophobic coating (TRSC) that addresses these challenges by synergistically integrating an intrinsically responsive triboelectric material (PTFE) with a hierarchical structure that both imparts superhydrophobicity and enhances electrical output. The coating is fabricated through a facile one‐step spray process using a suspension of PTFE precursor and surface‐modified SiO_2_ nanoparticles in DMF solvent, enabling rapid thorough drying within 90 s at a considerably low cost (<US$1 m^−2^). The resulting coating demonstrates comprehensive durability, including mechanical abrasion resistance (500 cycles), antichemical corrosion (pH 2–12), and high‐pressure tolerance (500 kPa), while maintaining consistent superhydrophobicity (contact angle 157°) and electrical performance. Moreover, TRSC exhibits universal substrate compatibility regardless of surface roughness and geometric complexity, enabling large‐scale (1.25 m × 1.25 m) deployment through automatic spraying systems. Under standardized testing conditions (4 cm × 4 cm), the coating achieves an open‐circuit voltage of 72 V and a short‐circuit current of 364 nA. The coating simultaneously functions as an effective triboelectric layer and a protective barrier, enabling versatile urban sensing applications through solid–solid contact (traffic monitoring), solid–liquid energy harvesting (raindrops), and noncontact sensing (human activity detection). This coating strategy offers a promising, scalable, cost‐effective approach for next‐generation self‐powered sensing networks.

## 2 Results and Discussion

### 2.1 Design Strategy and Working Mechanism of TRSC

**Figure 1:**
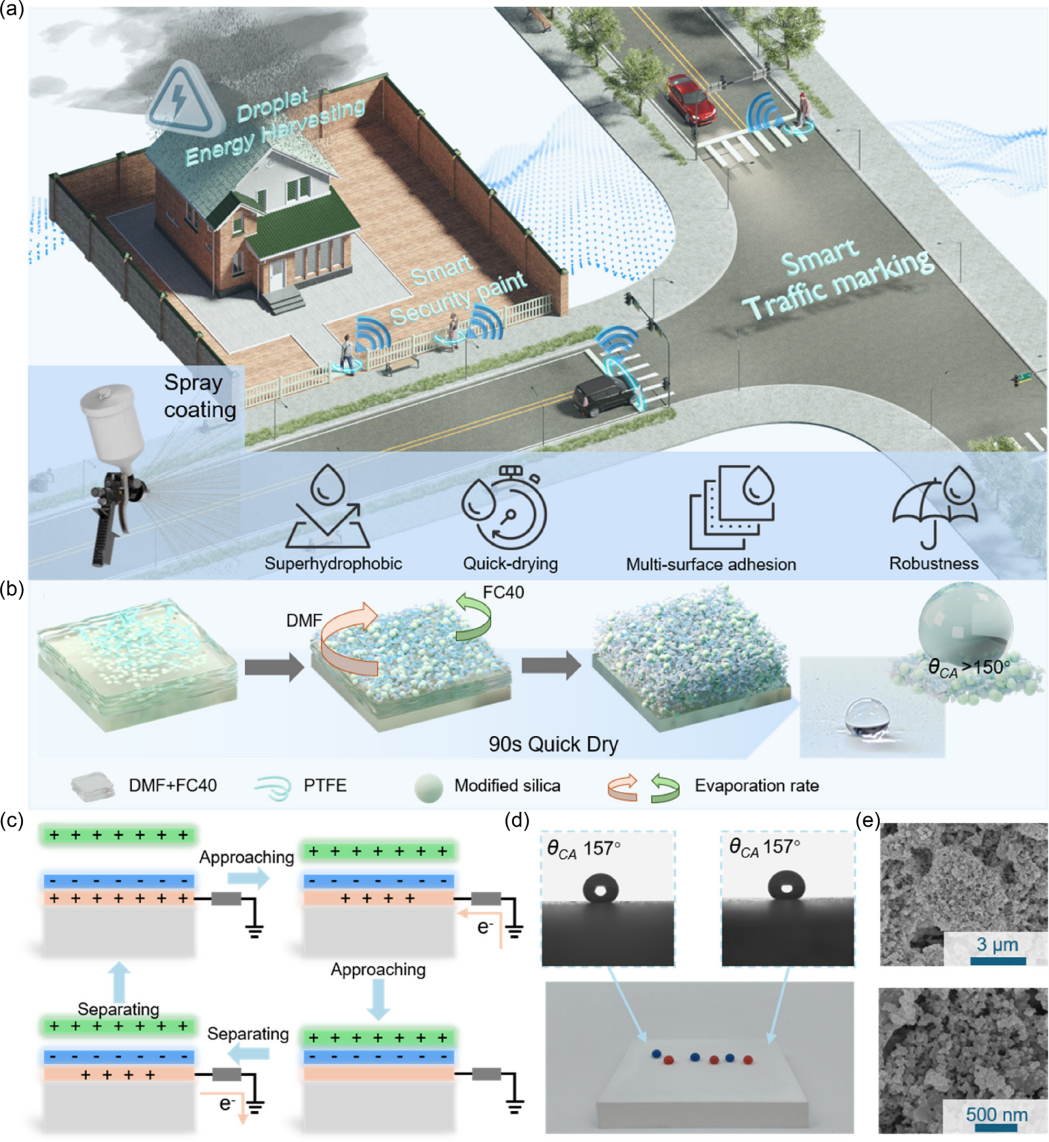
Applications, working principle, and superhydrophobic properties of TRSC. a) Schematic showing various TRSC applications for smart cities, including smart traffic marking on vehicle and pedestrian paths, security monitoring on barriers, and rain droplet energy harvesting. b) Schematic illustration of hierarchical superhydrophobic coating formation mechanism based on competitive evaporation process, showing the development of sponge‐like porous structures through differential evaporation of DMF and FC40. The photographs show the final coating's excellent water repellency. c) Working principle of triboelectric response of TRSC. d) Uniform superhydrophobicity demonstrated by dyed droplets maintaining spherical shapes on TRSC, with consistent contact angle at different locations. e) SEM images of the TRSC's hierarchical surface, showing the micron‐scale porous structure (main image) and the nanoscale roughness in the magnified view.

Smart city applications require extensive deployment of sensing networks in various urban scenarios, as illustrated in **Figure** **1**a, including traffic monitoring through road markings, security surveillance via barriers, and energy harvesting from rooftop raindrops. However, these outdoor applications face multiple challenges: the complex urban environment demands excellent water‐repellency and self‐cleaning capabilities; the diverse installation surfaces require universal adhesion regardless of material composition and surface geometry; and practical implementation needs quick‐drying and long‐term stability. To address these challenges, we develop a TRSC through a rational material design strategy. For the core functional component, we selected a PTFE precursor emulsion (AF1601), which serves a crucial dual role. Due to its highly electronegative carbon─fluorine (C─F) bonds, it acts as an excellent triboelectric‐negative material, while its formulation allows it to function as the primary binder for the coating. To complement this, surface‐modified SiO_2_ nanoparticles were separately dispersed in N,N‐dimethylformamide (DMF). These nanoparticles also fulfill two key functions: they act as a physical filler to enhance the coating's mechanical stability and wear resistance, and their surface chemistry is essential for creating the hierarchical structure during the solvent evaporation process. The subsequent mixing of these solutions enables the formation of a stable suspension that can be applied through a facile spray process (Figure S1, Supporting Information). This stable dispersion is primarily attributed to the addition of DMF, which acts as a crucial compatibilizer. It functions as a bridge between the perfluorinated FC40 solvent and the surface‐modified silica nanoparticles, preventing their aggregation and ensuring a homogeneous distribution throughout the final sprayable solution. The formation of the hierarchical micro/nanostructures and the rapid‐drying capability are governed by a competitive evaporation mechanism within the coating's binary solvent system. The formulation contains both DMF and Fluorinert FC40, which possess significantly different volatilities. Upon spraying, a repulsive interaction occurs between the amino groups of the KH550 surface modifier on the silica and the perfluorinated FC40 solvent. This repulsion selectively accelerates the evaporation rate of FC40, driving the formation of a porous, sponge‐like network as shown in Figure 1b. This porous architecture provides extensive pathways for the less volatile DMF to escape, enabling the entire film to dry thoroughly at room temperature. Simultaneously, the hydrophobic SiO_2_ nanoparticles act as heterogeneous nucleation sites, which accelerate PTFE crystallization and improve its crystallinity. This synergistic effect not only locks the porous structure in place but also contributes to the excellent wear resistance and superhydrophobicity of the coating. Moreover, the hydrophobic SiO_2_ nanoparticles act as heterogeneous nucleation sites, accelerating PTFE crystallization and improving its crystallinity. This synergistic effect enables room‐temperature curing while achieving excellent wear resistance and superhydrophobicity. The resulting coating can be directly deposited onto conductive electrodes to form single‐electrode TENGs, simultaneously achieving superhydrophobicity and triboelectric response.

The electrical response of TRSC‐based TENG relies on the coupling of contact electrification and electrostatic induction, as illustrated in Figure 1c. Initially, when the contact material is far from the TRSC surface, there is no significant interaction. As the material approaches, opposite charges are induced on the respective surfaces due to their different triboelectric properties, and to maintain charge balance, electrons migrate from the ground to the copper electrode. Upon physical contact, electrons transfer from the contact material to the TRSC surface due to their difference in electron affinity, establishing an electrical equilibrium. After separation, the negative charge on the TRSC surface induces a corresponding positive charge in the underlying copper electrode. This creates a potential difference that drives electrons to flow between the electrode and the ground, generating an alternating current signal through this cyclic process. For this electrical mechanism to function reliably in real‐world applications, the coating must be protected from environmental factors like moisture. This protection is provided by the TRSC's superhydrophobic nature, which originates from its unique hierarchical structure and the low surface energy of its PTFE component. As shown in Figure 1d, dyed water droplets maintain spherical shapes on the TRSC surface with a consistent contact angle *θ*_CA_ of 157° at different locations. When immersed in water, a clear silver mirror‐like reflection can be observed due to the trapped air layer between the coating surface and the water medium (Figure S2, Supporting Information). These surface structures, formed by the agglomeration of SiO_2_ nanoparticles within the PTFE matrix and combined with the low surface energy of the PTFE, lead to a stable Cassie‐Baxter state. Further analysis by energy‐dispersive X‐ray spectroscopy (EDS), shown in Figure S3, Supporting Information, confirms the presence and uniform distribution of both fluorine (from PTFE) and silicon (from SiO_2_) on the surface, verifying the effective integration of both key components.

### 2.2 Performance Characterization and Durability Assessment of TRSC

**Figure 2:**
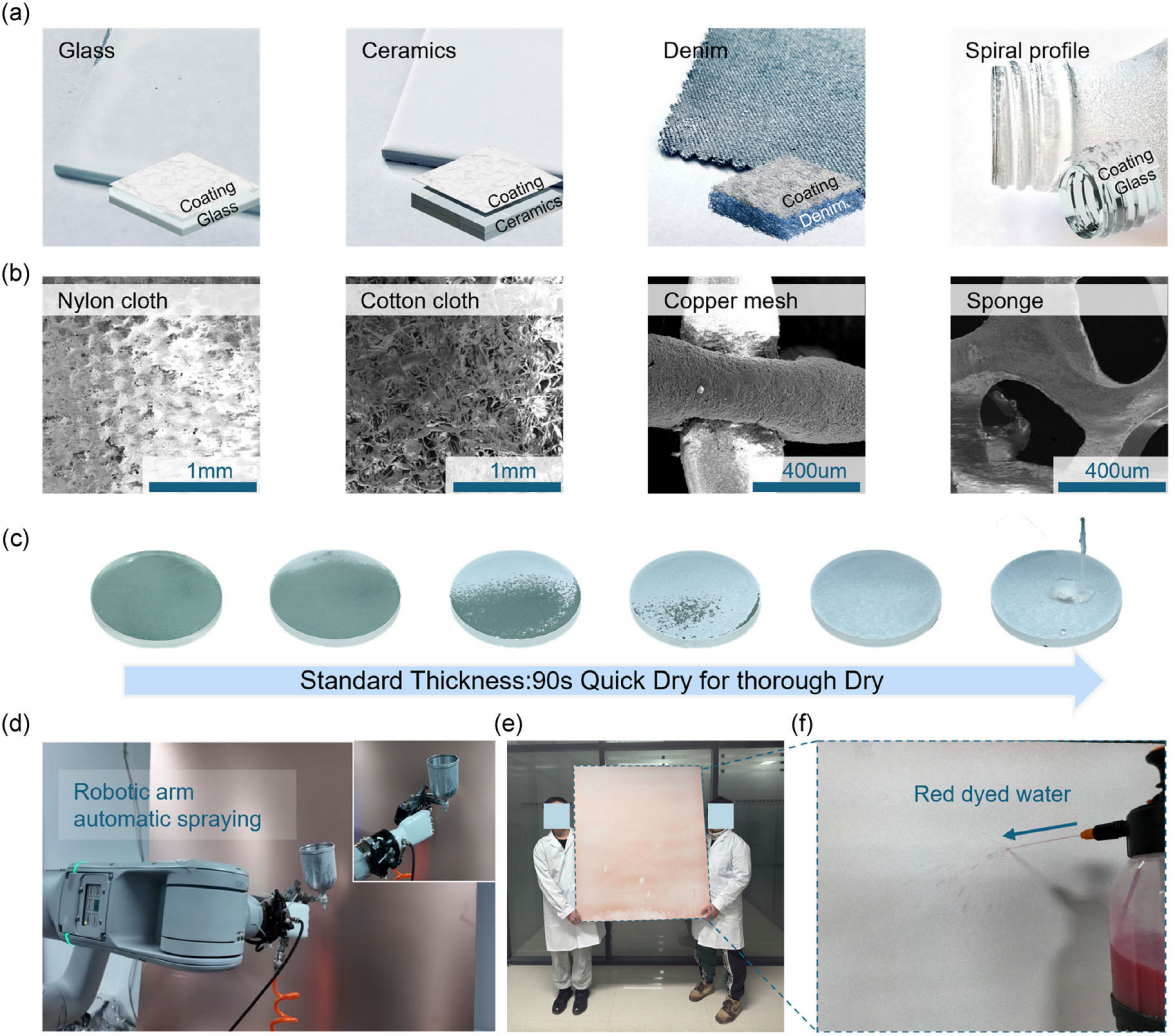
Universal applicability, rapid‐drying capability, and scalable fabrication of TRSC. a) Optical photographs showing the versatile adhesion of TRSC on a variety of substrates (flat glass, ceramics, textured denim fabric, and spiral‐profiled glass bottle). b) SEM images showing uniform coating morphology on various microstructured surfaces (nylon cloth, cotton cloth, copper mesh, and sponge substrates). c) Drying process of TRSC with thorough drying in 90 s at standard coating thickness. d) Demonstration of automated TRSC deposition using a robotic arm spraying system. e) Large‐scale TRSC coating (1.25 m × 1.25 m) on copper substrate, demonstrated by two adults (height: 175 cm) holding the coated panel. f) Sustained superhydrophobicity of the large‐scale coating after 6‐month environmental exposure.

The applicability of TRSC for practical large‐scale deployment is systematically evaluated through a series of tests. First, the coating's adhesion on various substrates through simple spray deposition is demonstrated in **Figure** **2**a. From flat glass and ceramics to textured denim fabric and spiral‐profiled glass bottles, TRSC forms uniform coverage regardless of substrate geometry. The excellent adhesion can be attributed to the optimized composition ratio between PTFE and surface‐modified SiO_2_ nanoparticles, where PTFE acts as an effective binder. This binding is further enhanced by the coating's rapid‐drying capability; The fast solidification, induced by the modified silica, is critical for preventing the liquid film from migrating or peeling before it cures, ensuring strong and uniform adhesion. Further microscopic examination by scanning electron microscopy (SEM) (Figure 2b and Figure S4, Supporting Information) reveals that this adaptability extends to substrates with complex microstructures, including nylon cloth, cotton cloth, copper mesh, and sponge materials, where the coating maintains intimate contact while preserving its essential microscale roughness for superhydrophobicity. Such universal adhesion originates from the rapid solvent evaporation process, which allows the coating to conform to various surface topographies before complete solidification. A crucial advantage of this coating strategy is its quick room‐temperature drying capability. As demonstrated in Figure 2c and Video S1, Supporting Information, the coating achieves quick thorough drying at standard thickness (50 μm) within 90 s under ambient conditions, with surface tack‐free time as short as a few seconds. This room‐temperature curing eliminates the need for additional heating equipment and enables immediate deployment after application, as evidenced by the instant water‐repelling behavior upon droplet contact. The efficient drying process also prevents coating deformation or cracking during solidification, ensuring coating integrity across different substrate geometries. The quick drying and simple processing characteristics enable scalable manufacturing, as demonstrated using a robotic arm automatic spraying system (Figure 2d), which successfully coated a large copper panel (1.25 m × 1.25 m) in vertical orientation without droplet accumulation or coating inhomogeneity (Figure 2e). Water jet tests conducted at various locations across the panel show consistent superhydrophobic performance, confirming uniform coating quality over a large area. The coating maintains stable performance after 6 months of environmental exposure (Figure 2f and Video S2, Supporting Information), indicating its long‐term durability under real‐world conditions.

**Figure 3:**
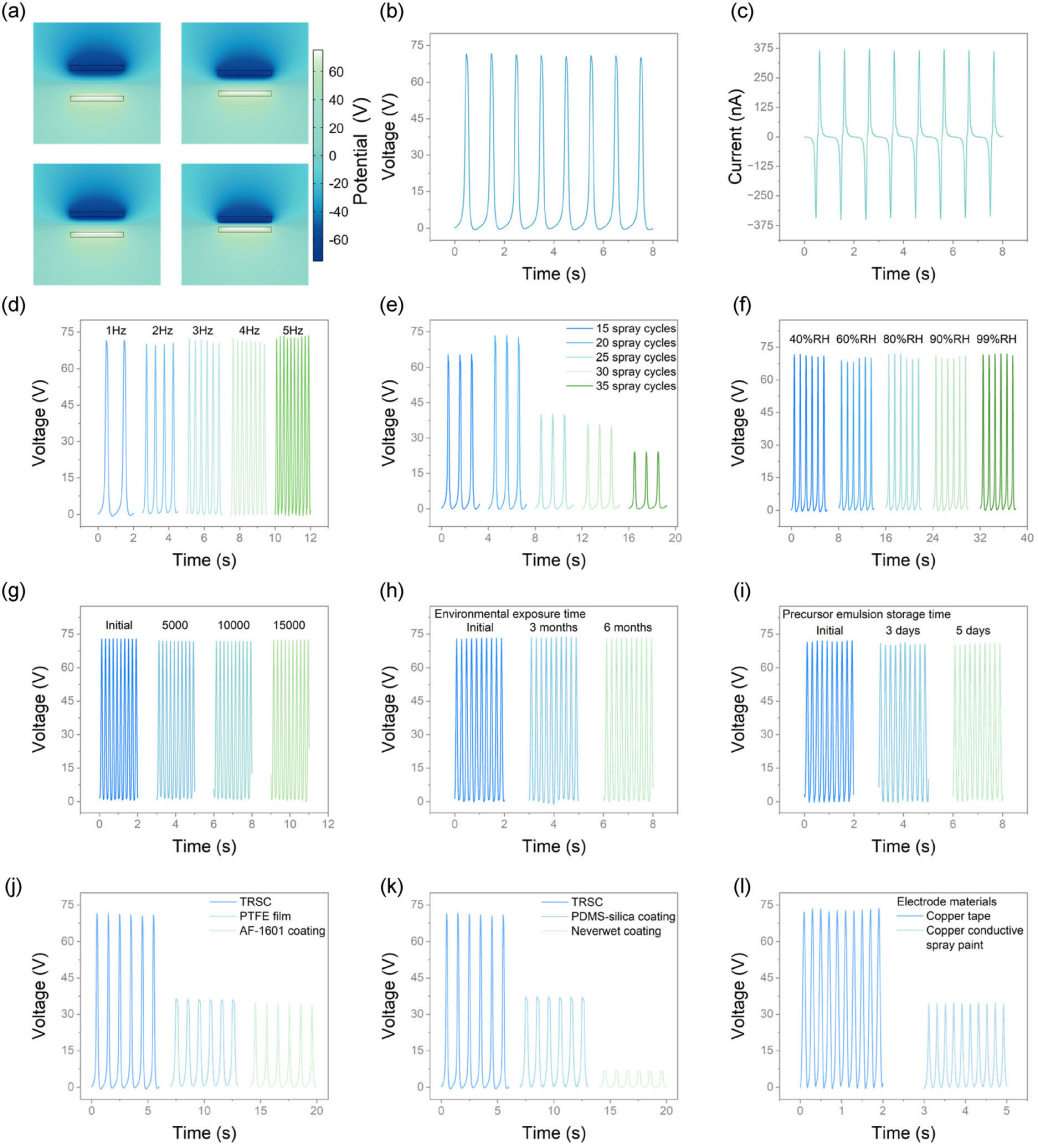
Electrical performance and stability characterization of TRSC‐based TENG. a) Simulation of electric potential distribution in TENG at different distances from the contact material. b) Open‐circuit voltage and c) short‐circuit current of TENG under standardized conditions (single‐electrode configuration, copper tape electrode with TRSC, 4 cm × 4 cm, nylon as contact material, 3 impacts with contact force of 3 N and at frequency of 1 Hz). d) Open‐circuit voltages at different frequencies under otherwise identical conditions. e) Open‐circuit voltages after different spray cycles of coating (15, 20, 25, 30, 35 cycles). f) Open‐circuit voltages under different relative humidity (RH) conditions (40%, 60%, 80%, 90%, 99% RH), showing consistent output. g) Durability test showing stable performance over 15 000 operation cycles (impact force of 3 N and frequency of 5 Hz). h) Robustness evaluation after 1, 3, and 6 months of environmental exposure. i) Influence of precursor emulsion storage time (1, 3, and 5 days) on the performance. j) Comparison of open‐circuit voltages with TRSC, commercial PTFE film, and AF1601‐coated PTFE film. k) Comparison of open‐circuit voltages with TRSC, laboratory‐prepared PDMS‐silica superhydrophobic coating, and commercial Neverwet superhydrophobic coating. l) Comparison of open‐circuit voltages between copper tape and copper conductive spray paint with TRSC, showing the potential of large‐scale manufacturing for smart city applications.

For electrical characterization, TRSC was spray‐coated onto a copper tape (4 cm × 4 cm) to construct a single‐electrode TENG. The electric potential distribution changes during contact‐separation cycles were analyzed through finite element simulation, as shown in **Figure** **3**a. Under standardized testing conditions (nylon as contact material, impact force of 3 N, and impact frequency of 1 Hz), the TENG exhibits an open‐circuit voltage of 72 V (Figure 3b) and a short‐circuit current of 364 nA (Figure 3c), demonstrating excellent electrical performance. The device maintains faithful signal patterns across different impact frequencies (1–5 Hz) as evidenced in Figure 3d, indicating reliable sensing capability. The coating thickness, controlled by the number of spray cycles, significantly influences the electrical performance. As shown in Figure 3e, the output voltage reaches its maximum of 72 V at 20 spray cycles (≈20 μm thickness). This optimal thickness reflects a balance in the surface structure formation: insufficient spray cycles (10 μm) result in incomplete micro/nanostructure development and reduced contact area, while excessive coating results in reduced porosity due to a slower solvent evaporation rate, both deteriorating the electrical performance of TENG. SEM images of TRSC processed with different spraying cycles are shown in Figure S5, Supporting Information. The TRSC‐based TENG demonstrates remarkable environmental stability, maintaining consistent output under RH ranging from 40% to 99% (Figure 3f) and showing stable performance over 15 000 times of continuous operation (Figure 3g). This exceptional humidity tolerance is attributed to the robust Cassie‐Baxter state established by the coating's unique porous structure. This structure traps a stable air layer that functions as an insulating shield, physically preventing ambient water molecules from contacting the triboelectric surface and condensing into a water film that shields the charge on the TRSC surface, and thereby minimizing charge dissipation. Such stability, combined with the coating's inherent ability to repel liquid water, is particularly crucial for outdoor applications such as rainy‐day energy harvesting and sensing in humid environments, where conventional TENGs often suffer from significant performance degradation. Furthermore, the inherent structural integrity of the TRSC, a composite of a PTFE binder and reinforcing SiO_2_ nanoparticles, suggests a high resistance against the physical collapse that can lead to wetting‐induced failure under prolonged condensation, which is a common weakness in more fragile systems. Notably, the coating exhibits excellent stability with sustained performance after 1, 3, and 6 months of environmental exposure (Figure 3h). In addition, its precursor solution maintains stability after storage for up to 5 days (Figure 3i), providing a sufficient time window for practical manufacturing and deployment processes.

To evaluate the advantages of TRSC, we compared its performance with several commercial and laboratory‐prepared materials. As shown in Figure 3j, TRSC exhibits an electrical output approximately twice that of conventional triboelectric materials like a flat PTFE film. This enhanced performance is attributed to the TRSC's hierarchical surface morphology; the micro/nanostructure created by the SiO_2_ nanoparticles significantly increases the effective surface area for contact electrification, allowing more charge to be generated and transferred in each cycle. When compared with other superhydrophobic coatings (Figure 3k), TRSC again significantly outperforms both a laboratory‐prepared PDMS‐silica coating and a commercial Neverwet coating. This superiority stems from two factors: first, the TRSC is based on the highly electronegative PTFE, a material inherently optimized for triboelectric performance, unlike materials like PDMS‐silica, which are chosen primarily for water repellency. Second, the unique sponge‐like structure of TRSC provides a larger effective contact area compared to the protruding microstructures often found in conventional superhydrophobic coatings. The coating also demonstrates promising performance when applied onto commercial copper conductive spray paint as an electrode (Figure 3i). Although the TENG fabricated with a sprayed copper paint electrode shows a lower output compared to one using copper tape, this is attributed to the inherent properties of the sprayed electrode. The paint's composite structure, consisting of copper particles within a polymer binder, results in lower intrinsic conductivity than solid copper foil, while minor process‐related nonuniformities can increase contact resistance. Furthermore, due to the excellent conformability of the TRSC, the interface it forms with the underlying layer is nearly perfect and free of voids or air gaps that can affect performance. This high‐quality interface means that the intrinsic resistance of the conductive layer itself becomes the dominant factor influencing the device's electrical output. Despite this, the all‐spray‐processed TENG maintains a decent output and offers significant advantages over conventional film‐based TENGs, namely its superior conformability to complex surfaces and its suitability for simple, large‐scale deployment.

**Figure 4:**
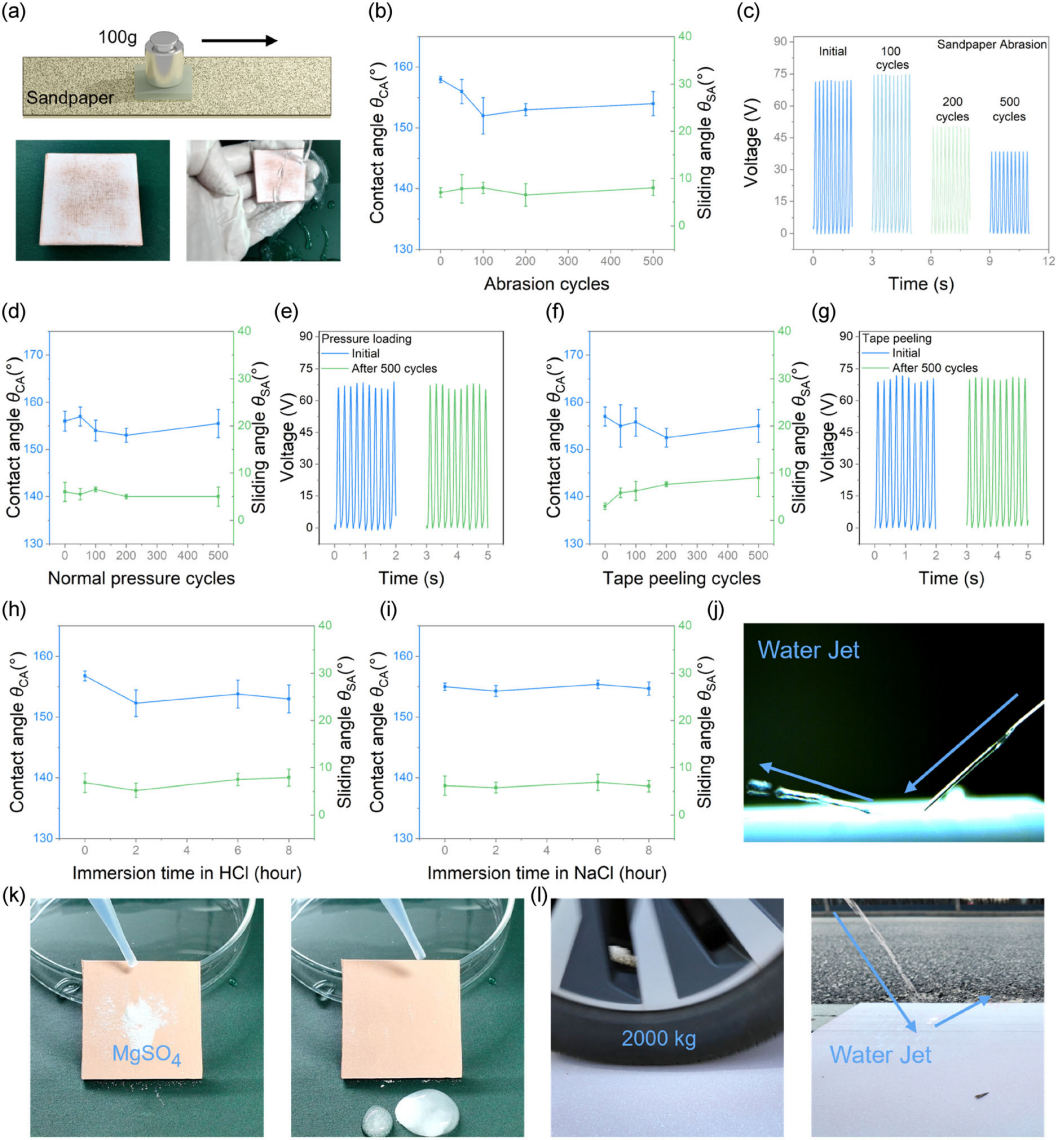
Robustness and durability of TRSC under various environmental conditions. a) Optical images demonstrating TRSC's water‐repelling capability after 500 abrasion cycles under a 100 g load on sandpaper, showing resistance to tap water impact. b) Contact angle and sliding angle measurements with increasing abrasion cycles under a 100 g load. c) Open‐circuit voltages after 500 abrasion cycles. d) Contact angle and sliding angle measurements with increasing cycles of normal pressure testing (500 kPa). e) Open‐circuit voltages after 500 pressure loading cycles. f) Contact angle and sliding angle measurements with increasing cycles of adhesive tape peeling. g) Open‐circuit voltages after 500 cycles of tape peeling. h) Contact angle and sliding angle measurements for immersion in hydrochloric acid (pH = 2). i) Contact angle and sliding angle measurements for immersion in saline solution (3.5‰ salinity). j) Optical image of water jet repelling, demonstrating maintained superhydrophobicity after hydrochloric acid immersion. k) Self‐cleaning performance demonstration using magnesium sulfate powder removal by water droplets. l) Optical image of water jet repelling, demonstrating maintained superhydrophobicity after vehicle rolling test.

The robustness and durability of TRSC under various environmental conditions are crucial for practical applications, where the protective layer may be exposed to mechanical wear, water impact, and dust accumulation. All durability tests were performed on TRSC coatings with optimal thickness (≈20 μm, as shown in Figure S6, Supporting Information). The mechanical robustness was first evaluated through sandpaper abrasion tests (1200‐grit) under a 100 g load. As shown in **Figure** **4**a and Video S3, Supporting Information, after 500 abrasion cycles, TRSC maintains its water‐repelling capability under tap water impact for one minute, simulating rain exposure. Postabrasion SEM imaging (Figure S7, Supporting Information) confirms that while the topmost surface exhibits some wear, the underlying porous morphology remains largely intact. The superhydrophobic property persists throughout the abrasion process, with contact angle (*θ*_CA_) ≈ 152.8° and sliding angle (*θ*_SA_) ≈ 8.3° after 500 cycles (Figure 4b). The electrical output, while showing some decrease in peak voltage after 200 and 500 cycles, remains stable, demonstrating mechanical abrasion tolerance (Figure 4c). The remarkable resilience of the superhydrophobicity is attributed to the coating's homogenous, bulk porous structure; mechanical abrasion simply exposes a new, underlying layer with nearly identical roughness. The moderate decrease in electrical output, however, is attributed to the partial flattening of the finest nanoscale surface features by the abrasive process. This reduces the effective contact area for charge generation, leading to a lower surface charge density. The coating's resilience under periodic high pressure was examined using a motorized platform for vertical force application. After 500 cycles of loading‐unloading under 500 kPa normal pressure, the superhydrophobicity persists (*θ*_CA _≈ 152°, *θ*_SA _≈ 8.4°) (Figure 4d), with consistent voltage output of TENG maintained throughout the process (Figure 4e). Additionally, tape‐peeling tests were conducted to evaluate the adhesion strength of the surface structure. The coating retains its superhydrophobicity (*θ*_CA _≈ 154.6°, *θ*_SA _≈ 9.2°) even after 500 peeling cycles (Figure 4f), with stable electrical performance (Figure 4g). The chemical stability of TRSC was verified through immersion tests in harsh environments commonly encountered in outdoor applications. After 8‐hour immersion in hydrochloric acid solution (HCl) (pH = 2) and saline solution (3.5‰ NaCl), simulating acid rain and marine environments, the coating maintains excellent water‐repellency (*θ*_CA_ ≈ 153.4°, *θ*_SA_ ≈ −8.9°) (Figure 4h,i). After HCl immersion, the robust water‐repelling capability is maintained, as demonstrated by the complete reflection of a high‐speed water jet (Figure 4j). The coating also exhibits excellent self‐cleaning properties, as evidenced by the effective removal of magnesium sulfate powder by water droplets (Figure 4k and Video S3, Supporting Information). Remarkably, when subjected to a vehicle rolling test (vehicle weight ≈2000 kg), the coating maintains its superhydrophobicity, with water jet being repelled immediately upon impact (Figure 4L and Video S3, Supporting Information), demonstrating its potential for real‐world traffic monitoring applications.

### 2.3 Applications of TRSC in Smart Cities

**Figure 5:**
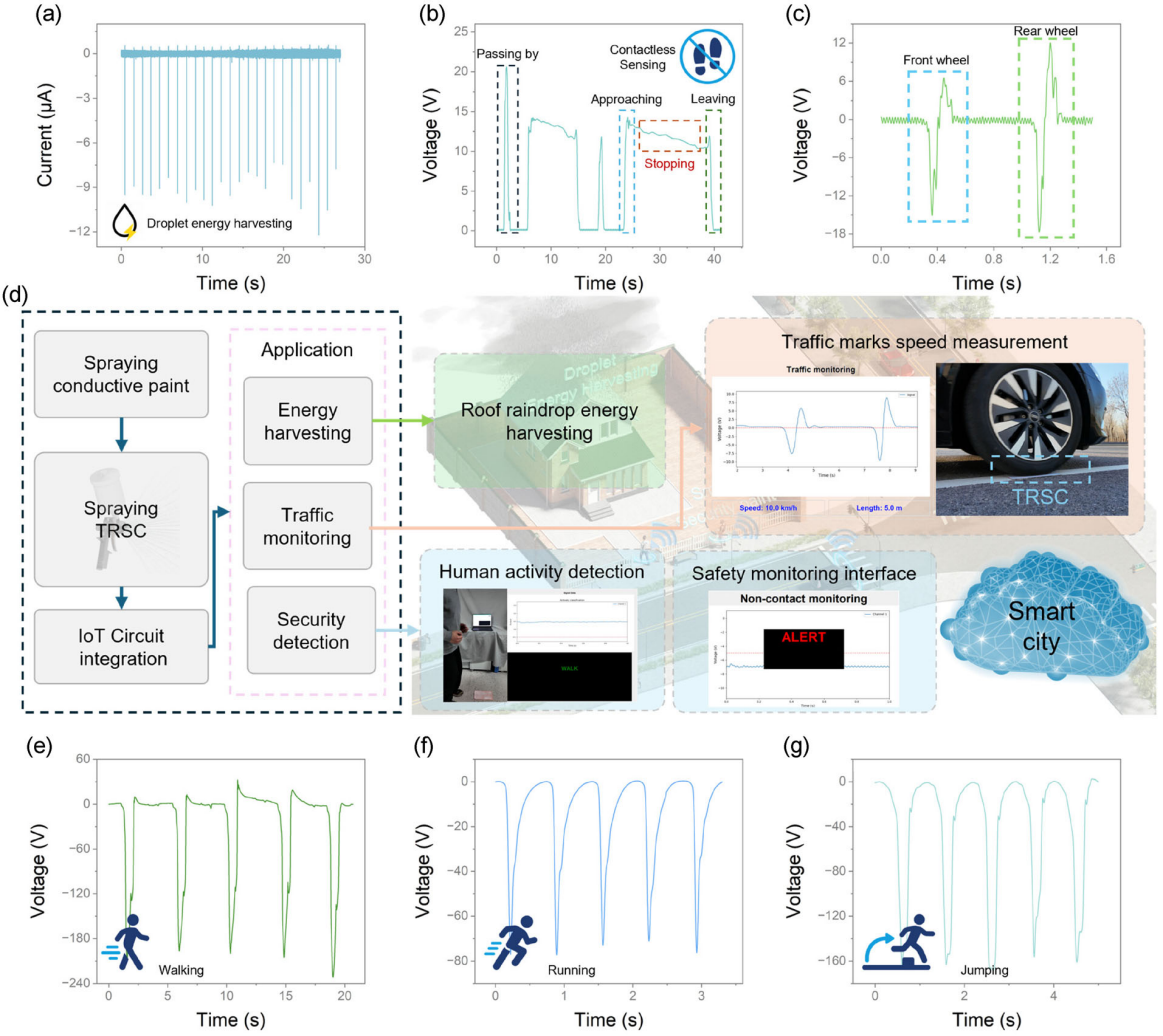
TRSC applications demonstrating robustness in smart city scenarios. a) Short‐circuit current generated by water droplets' impact on TRSC‐based TENG (4 cm × 4 cm active area, 50 cm falling height). b) Noncontact human motion detection signals (30 cm × 15 cm TENG) showing characteristic patterns during passing by, approaching, stopping, and leaving events. c) Optical images and corresponding output signals from vehicle rolling tests on TRSC‐coated road markings (30 cm × 15 cm TENG). d) IoT system integration demonstration, including spraying process, IoT circuit implementation, and multifunctional applications in energy harvesting, traffic measurement, and safety monitoring in smart city infrastructure. Characteristic output signals distinguishing different human activities: e) walking, f) running, and g) jumping.

To demonstrate the practical utility of TRSC in smart city applications, as shown in Figure 1a, comprehensive sensing demonstrations were conducted under various real‐world scenarios. For rainwater energy harvesting applications, water droplets impacting the TRSC‐based TENG (4 cm × 4 cm) from a height of 50 cm can generate well‐defined current pulses with an amplitude of short‐circuit current output of about 10 μA (**Figure** **5**a), demonstrating a stable electrical response even under liquid contact. The schematic is shown in Figure S8, Supporting Information. In this mechanism, the impacting droplet first becomes charged via contact electrification. As the droplet then spreads and retracts on the surface, it makes and breaks contact with a top electrode, and this cyclic connection drives a flow of electrons in the external circuit. This capability is particularly valuable for harvesting energy from natural precipitation on building surfaces while maintaining device functionality in wet conditions. The noncontact human detection capability was evaluated using a larger TENG (30 cm × 15 cm) to enhance the sensing range. During two independent passing by, approaching, stopping, and leaving cycles, the sensor generates distinct voltage patterns corresponding to different motion states (Figure 5b): characteristic peak signals during passing by, rapidly increasing voltage during approaching, relatively stable signals during stopping, and sharply decreasing voltage during leaving events. These unique electrical output features enable reliable human presence detection without direct contact, serving as a cost‐effective solution for security surveillance. The schematic is shown in Figure S9, Supporting Information. When implemented as contact‐based road markings, the TRSC coating exhibits both remarkable durability and precise sensing capabilities. The coating maintains consistent performance under repeated vehicle rolling, while generating real‐time voltage signals that clearly distinguish between front and rear wheel contacts (Figure 5c). This capability enables accurate measurement of vehicle speed and classification of vehicle types without requiring complex camera systems or embedded sensors, offering a simple yet effective solution for traffic monitoring. The schematic is shown in Figure S10, Supporting Information. The integration pathway toward smart city infrastructure is illustrated in Figure 5d, where an all‐spray‐processed fabrication approach is demonstrated. The sequential deposition of copper conductive spray paint electrode and TRSC coating to fabricate TENG, followed by IoT circuit integration, enables cost‐effective implementation for various applications. In addition, human activity recognition through the road markings reveals distinct electrical signatures: walking produces regular, high‐amplitude signals (≈180 V) with consistent frequency (Figure 5e), running generates lower‐amplitude pulses (≈70 V) with shorter intervals (Figure 5f), and jumping actions create distinctive peak patterns (≈160 V) corresponding to take‐off and landing phases (Figure 5g). These characteristic patterns demonstrate the coating's capability for precise activity classification, as demonstrated in Video S4, Figure S11, Supporting Information. In smart city applications, the diverse sensing capabilities enabled by the developed TRSC technology serve multiple functions: for energy harvesting, the coating enables efficient collection of environmental energy from raindrops on building surfaces and mechanical energy from human movements; for monitoring applications, it provides accurate traffic flow detection and speed measurement through road markings without complex infrastructure modification; and for security surveillance, the noncontact human detection and activity recognition capabilities offer reliable intrusion alerts and human behavior monitoring. Critically, the material's unique properties are key to reliable signal acquisition in these complex, real‐world environments. The coating's excellent superhydrophobicity, for example, ensures that signals from precipitation manifest as discrete, high‐frequency pulses by preventing the formation of a charge‐shielding water film. This makes them easily distinguishable from the low‐frequency, high‐amplitude signals generated by human or vehicular motion. This inherent signal separation, based on vast differences in signal characteristics, allows for straightforward filtering of potential cross‐talk between simultaneous events, ensuring high data integrity. This versatile coating strategy, combined with simple, fully spray‐based fabrication and IoT integration, provides a scalable approach for transforming conventional urban surfaces into intelligent interfaces, marking a significant step toward smart city implementation.

## 3 Conclusions

In summary, we have developed a robust TRSC that effectively addresses the key challenges in large‐scale deployment of self‐powered sensing networks. The coating exhibits consistent superhydrophobicity (contact angle 157°) and excellent electrical performance, delivering an open‐circuit voltage of 72 V, a short‐circuit current of 364 nA, and achieving a maximum instantaneous power density of 3.34 mW m^−2^ under optimal load. Furthermore, the TRSC demonstrates durability against chemical corrosion and resistance to mechanical abrasion, preserving substantial electrical output and stable superhydrophobic properties. The superhydrophobic coating exhibits enhanced triboelectric response while maintaining its functionality after 500 cycles of mechanical abrasion, tape‐peeling, compression tests, and chemical corrosion. Compared with conventional PTFE films and commercial hydrophobic coatings, our TRSC demonstrates superior triboelectric sensitivity and environmental stability, particularly maintaining over 95% of its initial performance under extreme humidity conditions (99% (RH)). More importantly, through a facile one‐step spray process, the coating achieves rapid thorough drying (90 s) in a general environment with material cost lower than US$1 m^−2^, enabled by manufacturing via automated spraying systems. Moreover, the compatibility with spray conductive paint enables all‐spray‐processed TENG fabrication. Both the coating and its precursor solution demonstrate excellent time stability over extended periods, fulfilling key requirements for commercial‐scale production. In urban environments, it functions effectively in solid–solid contact mode for traffic monitoring, solid–liquid interfaces for raindrop energy harvesting, and noncontact mode for human activity detection. The distinct characteristics in amplitude and frequency of the signals generated by these different events provide a strong basis for future development of signal processing algorithms to decouple composite signals in complex, multifunctional sensing scenarios. By integrating triboelectric and superhydrophobic functionalities into a single material system, this work provides a practical approach for transforming conventional urban surfaces into intelligent interfaces. Beyond the immediate applications demonstrated, this coating strategy opens new possibilities for constructing distributed sensing networks through simple surface modification, offering a scalable and economical solution for future smart city development.

## 4 Experimental Section

4.1

4.1.1

##### Materials

AF1601 emulsion was purchased from DuPont. *N,N*‐dimethylformamide (DMF) was obtained from Tianjin Damao Chemical Co., Ltd. Hydrophobic fumed silica nanoparticles (99%) were purchased from Macklin. Deionized water was produced by a laboratory ultrapure water system (Direct‐Pure UP‐10, RephiLe Bioscience). Hydrochloric acid (HCl), sodium chloride (NaCl), and magnesium sulfate (MgSO_4_) were purchased from Macklin Biochemical Co., Ltd. HCl and NaCl were used for chemical resistance evaluation, while MgSO_4_ was used for self‐cleaning performance assessment.

##### Preparation of TRSC

The TRSC was prepared through a facile spray‐coating process. F1601 emulsion (30 g) was dissolved in DMF (60 g) under magnetic stirring for 5 min. Separately, hydrophobic fumed silica nanoparticles (18 g) were dispersed in DMF (120 g) by ultrasonication for 10 min. The two solutions were then combined and stirred for an additional 10 min to form a homogeneous mixture. The resulting mixture was spray‐coated onto various substrates using a spray gun and allowed to dry at room temperature to form the superhydrophobic coating. Spraying parameters are shown in Table S1, Supporting Information.

##### Characterization and Measurement

Surface morphologies were examined by SEM (FEI Nova NanoSEM 450, USA). Static water contact angle (*θ*_CA_) and sliding angle (*θ*_SA_) measurements were conducted by depositing a 10 μL water droplet onto the sensor substrate and recording the results with a digital microscope (OSA100S‐T, Ningbo NB Scientific Instruments Co., Ltd., Zhejiang, China) at room temperature. The TENG was connected to electrometers (Model 6514, Keithley, USA), with signals transmitted to the computer using a synchronous data acquisition card (NI 6346, National Instruments).

The electrical performance under different humidity conditions was evaluated using a programable constant temperature and humidity test chamber (Y‐HD‐150 L, Hangtian Zhida, China). Tape peeling tests were performed by placing a steel cylinder (mass: 100 g) on the tape, applying direct pressure through continuous, periodic rolling motion to ensure uniform interaction between the tape and the prepared film. A motorized motion platform (PA050, Zolix Instruments Co., Ltd., China) was used to apply high normal forces (≈200 N) to the film with a periodicity of about 2 s. The normal force applied to the film was monitored in real‐time using a commercial force gauge (Leqing Handepai Instruments Co., Ltd., Zhejiang, China). The abrasion resistance was evaluated by fixing the film on the substrate and linearly pulling sandpaper (grit # 1200) along the film surface at a speed of ≈2 cm s^−1^ with a displacement of 5 cm as one abrasion cycle. A 100 g weight was placed on the sandpaper, and the resulting *θ*_CA_ and *θ*_SA_ were measured after different abrasion cycles to test the surface superhydrophobicity resistance. Chemical stability was assessed by immersing samples in hydrochloric acid solution (pH = 2) and saline solution (35‰ NaCl) for 8 h, followed by *θ*_CA_ and *θ*_SA_ measurements. Each reported value represents an average obtained from at least three repeated measurements under identical operational parameters. All data were processed and analyzed using OriginLab (Origin Pro 2016).

## Supporting Information

Supporting Information is available from the Wiley Online Library or from the author.

## Conflict of Interest

The authors declare no conflict of interest.

## Author Contributions

**Mingrui Wang**: conceptualization: (lead); methodology: (lead); software: (lead); visualization: (lead); writing—original draft (lead), **Ziyi Dai**: conceptualization: (equal); investigation: (equal); writing—original draft (equal), **Lining Zhang**: data curation: (supporting); visualization (supporting), **Tian Tang**: data curation: (supporting); investigation (supporting), **Kai Qian**: supervision (supporting), **Lihua Tang**: supervision: (lead); writing—review & editing (lead), **Kean C. Aw**: methodology: (supporting); supervision (supporting), **Zhiyi Wu**: supervision (equal); writing—review & editing (equal). **Mingrui Wang** and **Ziyi Dai** contributed equally to this work.

## Supporting information

Supplementary Material

## Data Availability

The data that support the findings of this study are available from the corresponding author upon reasonable request.
