# Supplementary material for: Robust, Scalable, and Triboelectric‐Responsive Superhydrophobic Coating for Versatile Smart City Applications
Source: Small Sci. 2025 Sep 13;5(11):2500387. doi: 10.1002/smsc.202500387 (PMC12622555; doi:10.1002/smsc.202500387)
Supplement: Supplementary file 1 — Supplementary Material [file SMSC-5-2500387-s001.zip › smsc202500387-sup-0001-SuppData-S5.pdf]

Supplementary Materials

of

Robust Triboelectric-Responsive Superhydrophobic Coating for Large-Scale Sensing Applications in Smart Cities

Mingrui Wang [†,1,2], Ziyi Dai [†,3], Lining Zhang[1], Tian Tang[2], Kai Qian[3], Lihua Tang [*,1], Kean C. Aw [1], Zhiyi Wu [*,2]

[1] Department of Mechanical and Mechatronics Engineering, The University of Auckland, Auckland, 1010, New Zealand

[2] Beijing Institute of Nanoenergy and Nanosystems, Chinese Academy of Sciences, Beijing 100083, China

[3] School of Integrated Circuits, Shandong University, Jinan 250100, China

† The authors contribute equally to this work.

* Corresponding authors: l.tang@auckland.ac.nz (Tang); wuzhiyi@binn.cas.cn (Wu)

The PDF file includes:

- Supplementary **Figures S1** to **S11**
- Supplementary **Table S1**
- Descriptions of Supplementary **Videos S1** to **S4**

Supplementary Figures

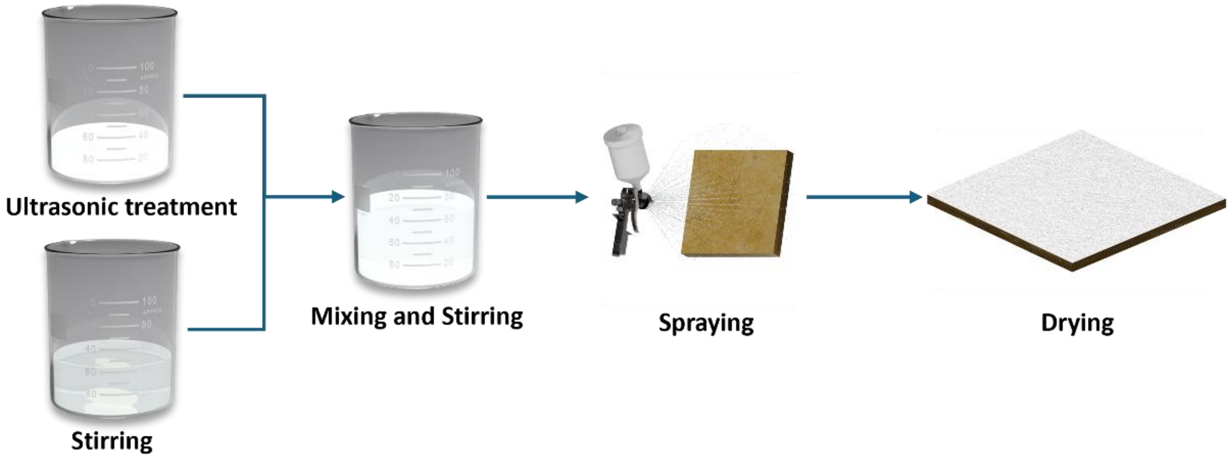

Figure S1. TRSC preparation through a facile spray process.

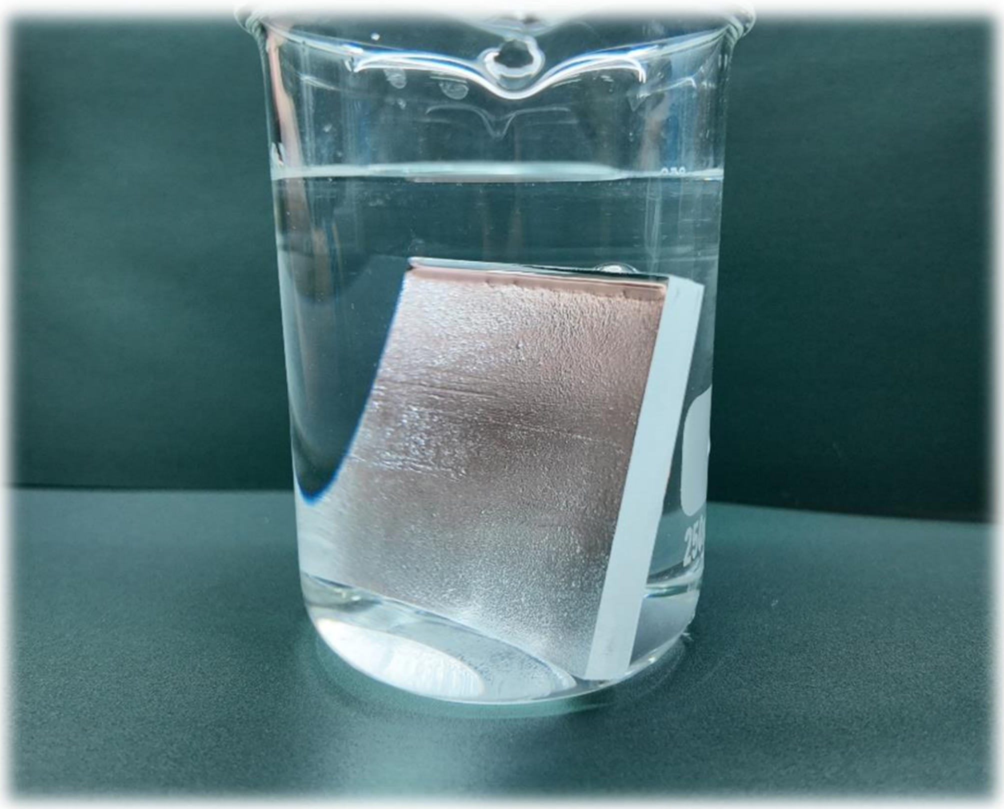

Figure S2. Clear silver mirror-like reflection of TRSC.

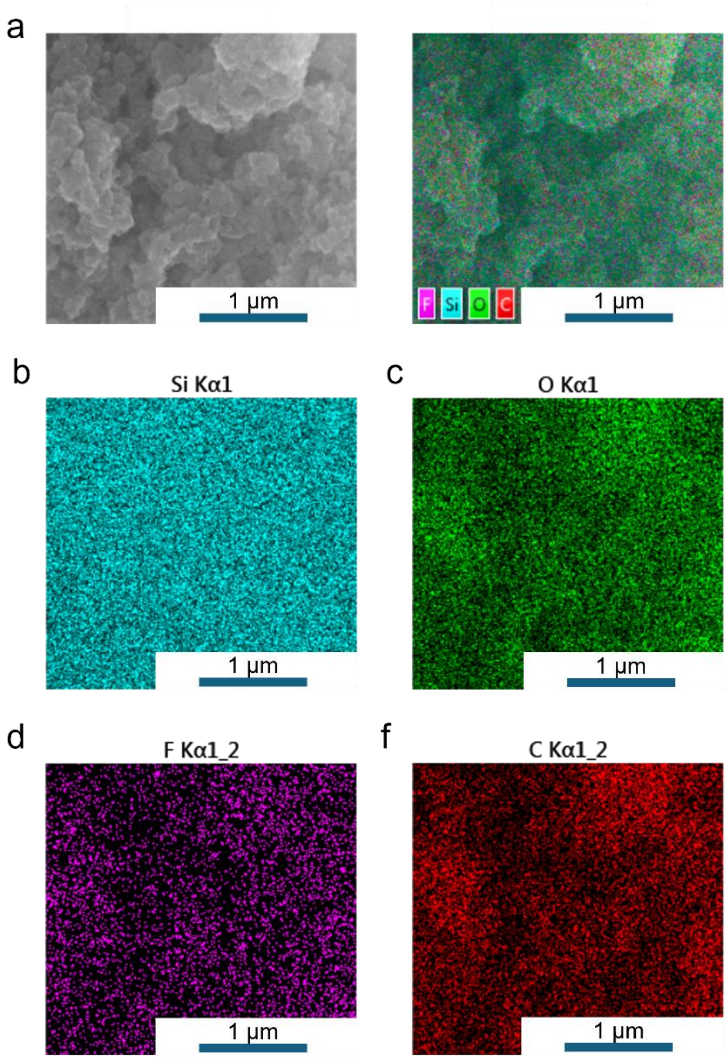

Figure S3. EDS elemental mapping of the TRSC surface. (a) SEM image of the analyzed area. (b-f) Corresponding elemental maps for Silicon (Si), Oxygen (O), Fluorine (F), and Carbon (C).

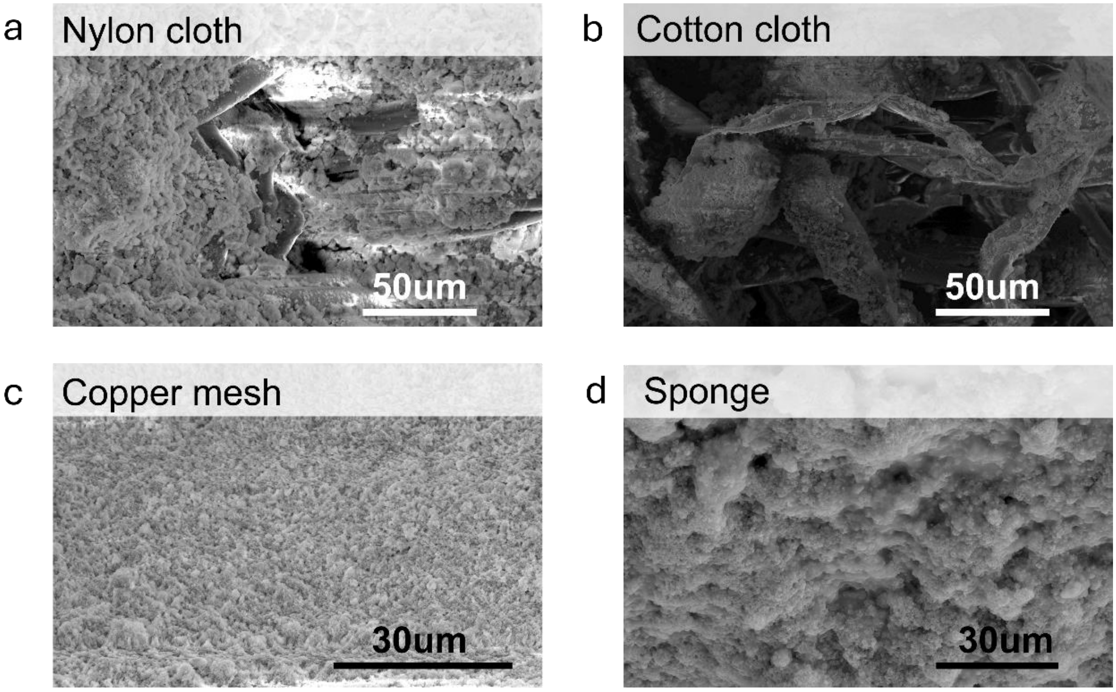

Figure S4. High resolution SEM images showing uniform coating morphology on various microstructured surfaces: **a**. Nylon cloth, **b**. Cotton cloth, **c**. Copper mesh and **d**. Sponge substrates.

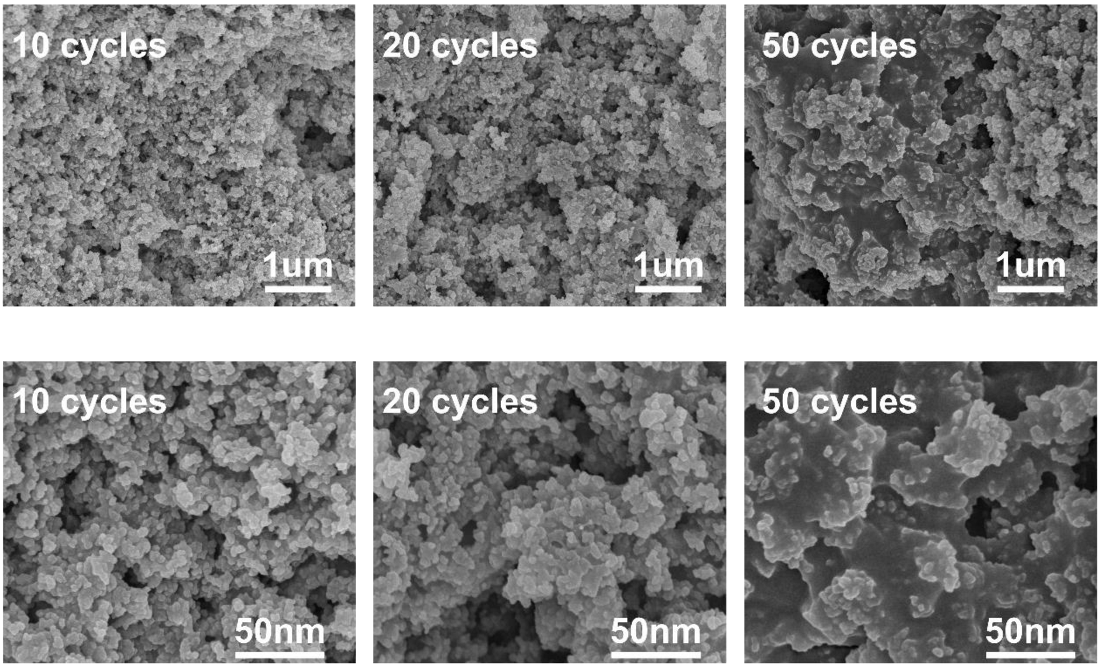

Figure S5. SEM images showing TRSC processed with different spray cycles.

As shown in **Figure S5**, when the spraying cycle is less, the three-dimensional micro-nano structure is sharper due to the different solvent evaporation rates. While with extensive spraying cycles, the PTFE precursor will agglomerate silica due to the slow solvent evaporation and cannot form a nanostructure. Thus, the relative surface area decreases, resulting in the reduced electrical output.

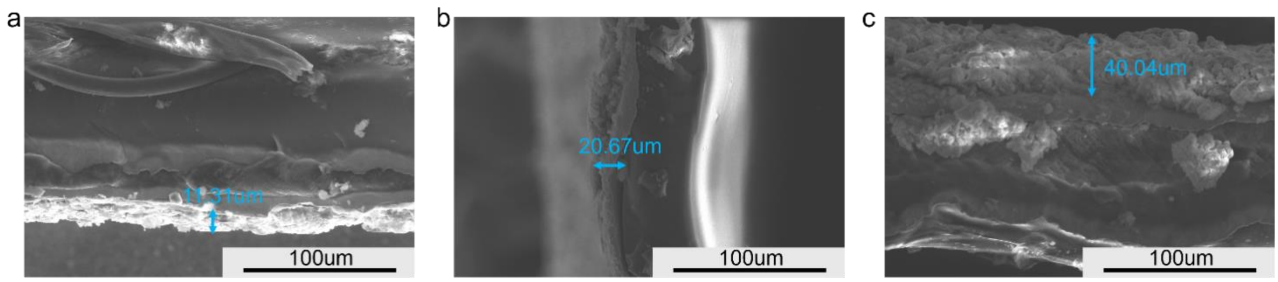

Figure S6. Cross-sectional SEM images showing the coating thickness of TRSC prepared with **a**. 15 spray cycles (~11.3 μm), **b**. 20 spray cycles (~20.7 μm), and **c**. 35 spray cycles (~40.0 μm).

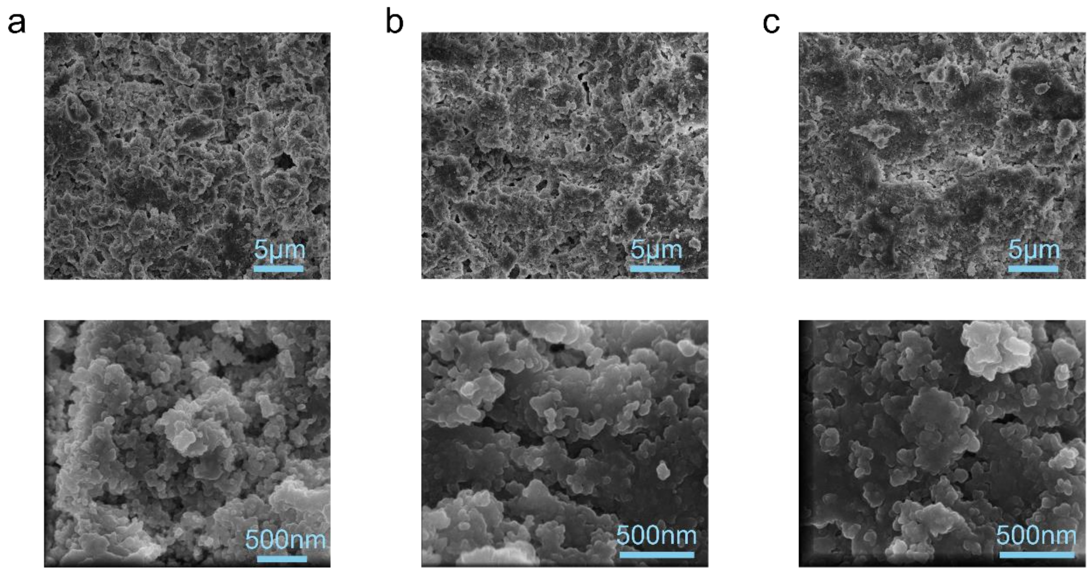

Figure S7. SEM images of the TRSC surface after abrasion tests using 1200-grit sandpaper with a 100 g load, shown after (a) 100, (b) 200, and (c) 500 cycles.

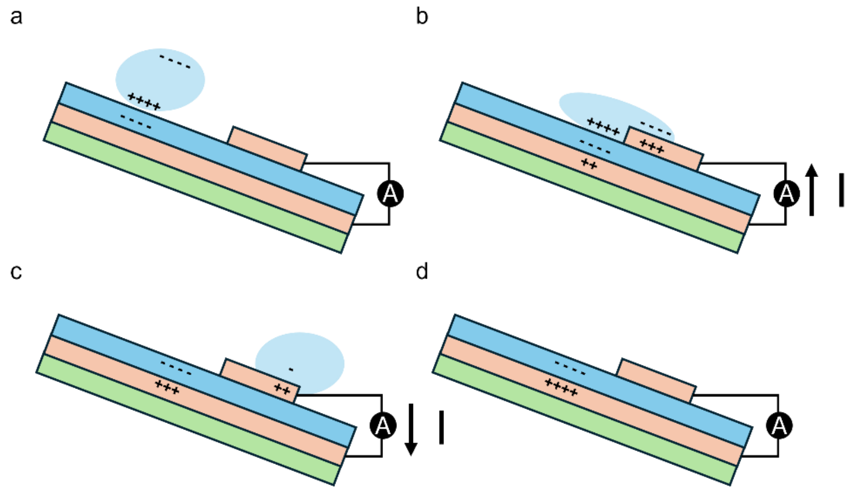

Figure S8. Working principle of the TRSC-based droplet TENG. **a**. A droplet lands on the TRSC surface and becomes positively charged due to contact electrification. **b**. As the droplet spreads, it makes contact with the top Cu electrode, forming an electrical connection that drives a current. **c**. The droplet then retracts and detaches from the electrode, causing a reverse current flow as the charge equilibrium shifts. **d**. Finally, the droplet slides off the superhydrophobic surface, and the

system returns to its initial state, ready for the next cycle.

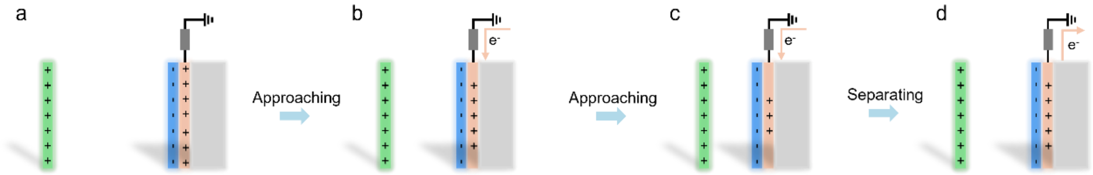

Figure S9. Working principle for non-contact human detection. a. Initial state with a person at a distance. b-c. As the person approaches, electrostatic induction drives a current. d. A reverse current is generated as the person moves away.

For non-contact sensing, the TENG is prepared on a surface like a wall. The mechanism relies on electrostatic induction between a person and the sensor. Initially, the TRSC maintains a stable negative surface charge, and a person is at a distance. As a typically positively charged human body approaches, its electric field partially shields the TRSC's field. To maintain electrostatic equilibrium, electrons flow into the electrode. As the person gets closer, the shielding effect intensifies, and more electrons flow into the electrode. When the person moves away, the shielding effect vanishes, and the electrons flow back out of the electrode to re-balance the TRSC's surface charge. This movement of electrons in response to the person's proximity generates the detection signal.

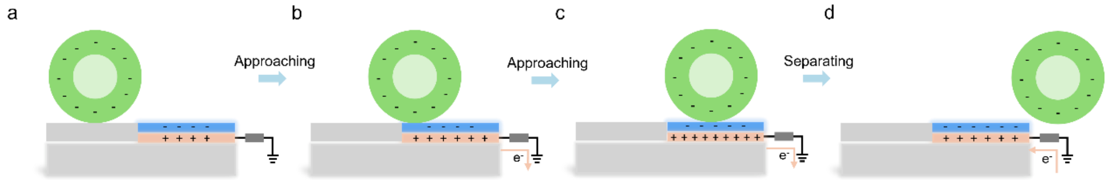

Figure S10. Working principle for traffic monitoring. a. Initial state before contact. b-c. A tire makes contact and passes over the sensor, inducing a current flow. d. The tire leaves, causing a reverse current flow as the sensor resets.

For traffic monitoring, the TENG is fabricated by spraying a conductive electrode and then the TRSC layer onto a non-conductive road marking. Initially, the TRSC has a negative surface charge, which is balanced by positive charges in the electrode. A car tire is also highly electronegative due to constant friction with the ground. When the more-electronegative tire begins to make contact with the TRSC, its strong negative electric field induces a large positive charge in the underlying electrode, causing electrons to flow out of the electrode. As the tire fully rolls over the sensor, this induction effect is maximized. When the tire leaves the TRSC surface, the induction effect is removed and the system's charge re-balances, causing electrons to flow back into the electrode. This cyclic process generates the sensing signal for each wheel pass.

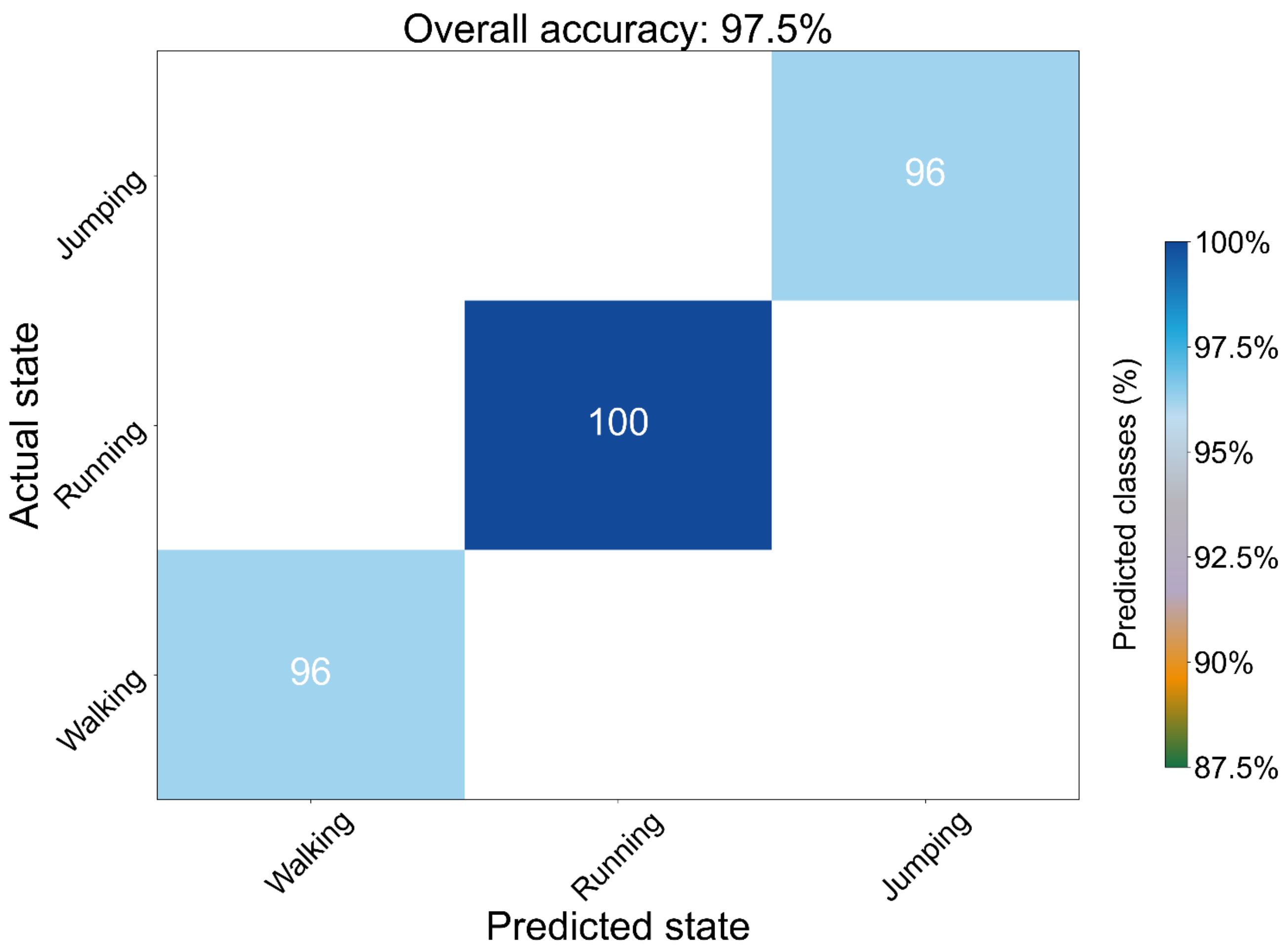

Figure S11. Deep learning confusion matrix for human activity detection.

This study utilizes a one-dimensional convolutional neural network (1D CNN) for the three-class classification of univariate time series with a length of 1500 steps. The network architecture consists of three convolutional blocks; the first block uses 32 filters (kernel size 7, ReLU activation), while the subsequent two blocks use 64 filters (kernel size 7, ReLU activation). Each convolutional layer is followed by a max-pooling layer (MaxPooling1D) with a pool size of 2 to reduce sequence length. After the convolutional blocks, a Global Average Pooling layer (GlobalAveragePooling1D) is employed to aggregate features by averaging across the time dimension for each filter, significantly reducing model parameters. Finally, a dense output layer with 3 nodes utilizes a Softmax activation function to produce class probabilities. For training, the Adam optimizer was used with a batch size of 32, running for 20 epochs with EarlyStopping monitoring validation loss to prevent overfitting. The model was implemented using Python with the pytorch library and trained on a device equipped with an NVIDIA GeForce RTX 3080 Laptop GPU.

Table S1

Parameter	Value
Spraying Motion	10 cm back-and-forth pass
Cycle Definition	1 back-and-forth pass
Cycle Duration	~0.5 s
Spraying Distance	20 cm
Spraying Angle	45°
Spraying Pressure	0.6 MPa
Solution Volume per Cycle	~0.2 mL

Supplementary Videos

Video S1. Drying process of TRSC with thorough drying in 90 seconds at standard coating thickness. The substrate is acrylic and the ambient temperature is 15 °C.

Video S2. Verification of superhydrophobicity of large-scale coating after 6-month environmental exposure.

Video S3. Robustness and durability of TRSC:
(a) Demonstration of maintained superhydrophobicity of TRSC after 500 cycles of sandpaper abrasion, demonstrated by water repelling under tap water impact.
(b) Demonstration of self-cleaning ability of TRSC, demonstrated by removal of magnesium sulfate powder with droplets.
(c) Demonstration of maintained superhydrophobicity after wheel rolling, demonstrated by water jet repelling.

Video S4. Demonstration of TRSC-based human activity detection, distinguishing among walking, running, and jumping.
